# Immunotherapy With Human Gamma Delta T Cells—Synergistic Potential of Epigenetic Drugs?

**DOI:** 10.3389/fimmu.2018.00512

**Published:** 2018-03-13

**Authors:** Jaydeep Bhat, Léonce Kouakanou, Christian Peters, Zhinan Yin, Dieter Kabelitz

**Affiliations:** ^1^Institute of Immunology, University of Kiel, Kiel, Germany; ^2^The First Affiliated Hospital, Biomedical Translational Research Institute, Guangdong Province Key Laboratory of Molecular Immunology and Antibody Engineering, Jinan University, Guangzhou, China

**Keywords:** Bromodomain and ExtraTerminal domain, checkpoint inhibitors, DNA methylation, gamma delta T cells, histone acetylation, immunotherapy, natural-killer group 2 member D, programmed death 1

## Introduction

Epigenetics has emerged as one of the fastest growing concepts, adding more than 45 new publications every day, spreading through various fields (1). Conrad Waddington coined the term “epigenetics” in 1942; however, a multitude of definitions has been endorsed by different researchers. In essence, Waddington’s definition of “epigenetics” and its redefinition by Holiday is at the heart of cellular function. Hence, it is obvious that epigenetic regulation plays a central role also in the specification, differentiation, and functional plasticity of T lymphocytes (2). T-cell fate decision in progenitor cells, functional CD4 T-cell plasticity, CD8 T-cell differentiation, but also T-cell memory, are all substantially governed by epigenetic mechanisms (3–7). Here, we focus on the current development of drugs targeting major pathways of epigenetic regulation and their possible impact on γδ T-cell multifunctionality. We aim to develop concepts of how some of these approaches might help to improve the efficacy of γδ T-cell-based immunotherapies.

**Figure 1:**
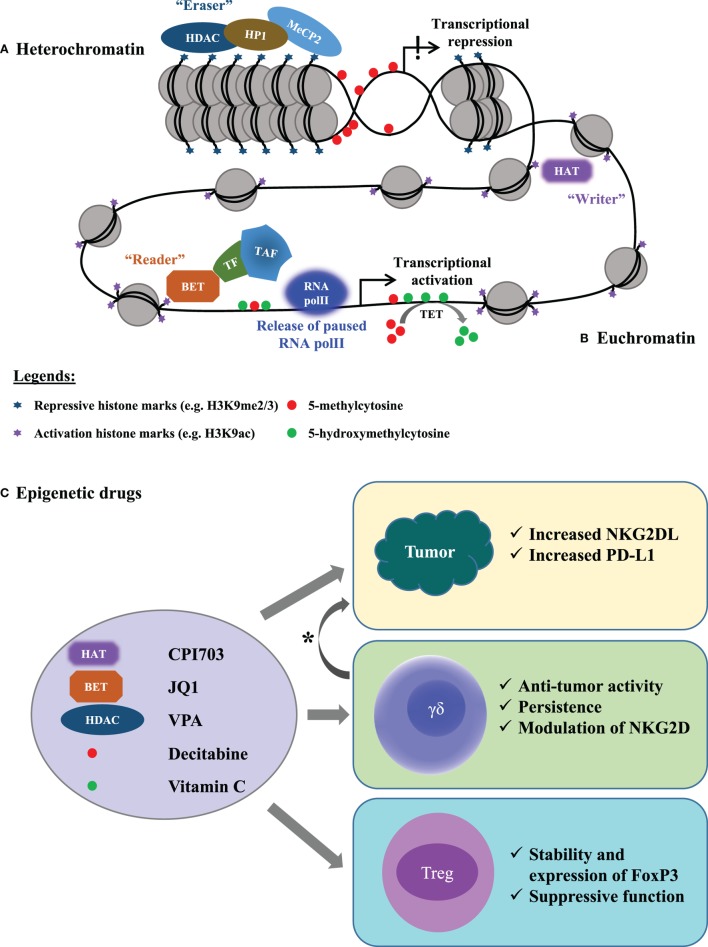
An overview of epigenetic mechanisms governing cellular processes and the drugs targeting respective epigenetic processes. There are two possible states of chromatin organization: **(A)** the “closed” chromatin associated with heterochromatin formation and transcriptional repression drives gene silencing. **(B)** The mechanistic organization of euchromatin maintains the “open” chromatin confirmation and allows active gene expression. **(C)** Examples of how epigenetic drugs modulate the γδ T-cell/Treg/tumor interaction. In the circle (left part), the epigenetic drugs (on the right-hand side) that are either in pre-clinical development or clinically approved are listed along with the respective target proteins (on the left-hand side). These are the key proteins for diverse epigenetic processes. The effect of the listed epigenetic drugs on immune cells (γδ T cells and Treg) and tumor cells are shown in the rectangles (right part). As marked by asterisk (*), the epigenetic drugs are proposed to synergize, leading to increased efficacy of γδ T cell-based immunotherapy. HDAC, histone deacetylase; HP1, heterochromatin protein 1; MeCP2, methyl-CpG binding protein 2; HAT, histone acetyltransferase; BET, Bromodomain and ExtraTerminal; TF, transcription factor; TAF, transcription-associated factors; RNA polII, RNA polymerase II; TET, ten-eleven translocation; VPA, valproic acid; NKG2D, natural-killer group 2, member D receptor protein; NKG2DL, ligands for NKG2D receptor protein; PD-L1, programmed death ligand 1; FoxP3, forkhead Box P3; Treg, regulatory T cells.

The dynamic construction of chromatin organization exists in two principal states, i.e., transcriptionally repressive “heterochromatin” and active “euchromatin.” The heterochromatin formation (Figure 1A) is mediated by SET domain, the chromodomain, and plant homeodomain finger, found in the heterochromatin protein 1 (HP1)/chromobox, and the chromodomain helicase-DNA-binding subfamilies, recognizing histone methylation (e.g., H3K9 di- and tri- methylation) (8). Histone deacetylase (HDAC) associates with HP1, then recognize histone methyltransferases and methylated DNA *via* methyl-binding proteins such as MeCP2. HDACs also interact with DNA methyltransferases (DNMTs; the enzymes catalyze DNA methylation), thus forming the regulatory axis of a multiprotein complex responsible for transcriptional repression (9–11). DNA demethylation can be achieved “actively” by the hydroxylation of 5-methylcytosine to 5-hydroxymethyl cytosine mediated by the ten-eleven translocation (TET) enzymes (12, 13). In contrast, the “euchromatin” formation is a complex, multistep process involving post-translational modifications (PTM) of histones and also chromatin-remodeling complex (Figure 1B). In addition to other PTM, the acetylation of histone leading to the “euchromatin” formation has already been reported during the 1990s (14). This process of histone lysine acetylation is mediated by HAT and is recognized by the bromodomain (Brd) proteins, additionally recruiting proteins. The Brd proteins are thus categorized as components of HAT complexes, components of chromatin-remodeling complexes, and Brd and ExtraTerminal domain (BET) proteins. BET proteins, particularly Brd2 and Brd3, play a multifaceted role by maintaining euchromatin status and simultaneously “reading” both acetylated histones and transcription factors. By recruiting and coupling the transcriptional machinery to the target gene promoter and/or enhancer sites, BET proteins further release paused RNA polymerase II for the respective gene activation (15–19). Additionally, the proteins involved in the principal states of chromatin organization have multiple functions including enzymatic activity. Such an important complexity in protein/enzyme function provides a leverage for the epigenetic drugs.

Nonetheless, it is important to realize (but currently not yet a major focus of epigenetic research) that any enzymatic activity (and thus epigenetic regulation) depends on the appropriate cellular metabolism. While the central role of the cellular metabolism for the maintenance of stem cell pluripotency (which is drastically influenced by epigenetics) is well known, the respective roles of metabolic pathways and nutrients availability versus epigenetics for the differentiation and plasticity of immune cells have only recently been appreciated (20, 21).

## Epigenetic Drugs

In view of the central role of epigenetic regulation for developmental biology and cellular activation, proliferation, and differentiation, it comes as no surprise that many drugs targeting specific steps of epigenetic regulation have been developed (Figure 1C). If suitable for clinical application, such drugs might have broad applications for the treatment of (certain types of) cancer but also autoimmune and chronic inflammatory diseases.

Currently, two hypomethylating agents targeting epigenetic “erasers,” decitabine (5-aza-2′-deoxycitidine) and azacitidine (5-azacitidine) are approved by the US Food and Drug Administration (FDA) for the treatment of myelodysplastic syndromes, but are also used in other clinical conditions (22). The major effect of such agents is to induce hypomethylation of CpG islands thereby allowing re-expression of suppressed genes including tumor suppressor genes. Not unexpectedly, hypomethylating drugs have major effects on immune cells including the stabilization of FoxP3 expression and Treg activity (23). In addition, numerous studies have investigated effects of hypomethylating agents on NK cells, dendritic cells, and T cells [see Ref. (22)]. It is difficult to draw general conclusions as the reported effects may be linked to specific experimental conditions or treatment regimens, but immunomodulatory effects are quite obvious (22). Immunogenicity of tumors might increase due to re-expression of tumor-associated antigens. However, hypomethylating agents might also promote tumor resistance through upregulation of inhibitory molecules like PD-1 and/or PD-L1 (24, 25). Obviously, the complexity of the effects of epigenetic drugs needs to be carefully evaluated. A major breakthrough in cancer immunotherapy has been the introduction of checkpoint inhibitors into clinical practice. Currently, several trials have been initiated where azacitidine is combined with PD-1/PD-L1 or CTLA-4 checkpoint inhibitors in hematological malignancies and colorectal cancer (26). Another regulator of DNA methylation is Vitamin C (VC). In addition to its antioxidant activity, VC also activates TET enzyme activity and thereby promotes 5-hydroxymethylation of DNA (27, 28).

Like hypomethylating agents, HDAC inhibitors (HDACi) have multiple effects on tumor cells but also on immune cells. In fact, their therapeutic efficacy against cancer is likely to depend on the simultaneous modulation of the immune system (29). Several structural classes of HDCAi have been developed. While some HDACi inhibit all HDACs, others are specific for class I and class IIa HDACs (e.g., valproic acid, VPA) or only class I HDAC (e.g., entinostat). Some HDACi including VPA upregulate the expression of NKG2D ligands on tumor cells and thereby augment the susceptibility to recognition and lysis by NK cells and γδ T cells (30, 31). As of today, several HDACi have been approved by the FDA either as monotherapy or in combination with other drugs, such as with PD-1 or CTLA-4 checkpoint inhibitors (26), for the treatment of hematological malignancies and some solid tumors [see Ref. (26)].

Epigenetic drugs which target epigenetic “readers” are BET inhibitors. The inhibition of BET proteins has a broad impact on gene regulation and may have a therapeutic effect in cancer (18). JQ-1, a pan-BET inhibitor blocks Th17 differentiation and thereby suppresses Th17-related inflammatory diseases in mouse models (19, 32). Importantly, recent studies point to a selective effect of JQ-1 on PD-L1 expression. PD-L1 is a direct target gene of the BET family member Brd4, and BET inhibition by JQ-1 has been found to enhance anti-tumor immunity by suppressing the PD-L1 expression on tumor cells and antigen-presenting cells but also through upregulation of NKG2D ligand MICA on tumor cells (33–35). BET inhibition also affects T-cell differentiation. A recent study reported superior *in vivo* persistence and anti-tumor activity of tumor antigen-specific murine T cells upon adoptive transfer (36). Moreover, BET proteins appear to be interesting targets for synergistic anti-tumor effects in combination with other inhibitors targeting, e.g., PI3-kinase (37), Bcl-2 (38), PARP (39), or HDAC (40). Last but not least, BET inhibitors like JQ-1 might also synergize with checkpoint inhibitors to facilitate efficient anti-tumor immune responses (41). Based on promising pre-clinical results, BET inhibitors have entered clinical trials. However, many details of how BET inhibitors work at the molecular level and which cells and tissues are differentially affected, are not yet precisely known; therefore, the adverse side effect profile of various BET inhibitors needs to be studied in detail (42).

## Plasticity of γδ T Cells

γδ T cells are considered to link innate and adaptive immunity because they can be rapidly activated *via* their T-cell receptor (TCR) in an MHC-independent manner (e.g., recognition of pyrophosphates in the case of human Vγ9Vδ2 T cells) but also express functional innate receptors such as toll-like receptors (43). Importantly, human Vγ9Vδ2 T cells cannot only differentiate into different cytokine-producing subsets, but also may acquire regulatory activity and “professional” antigen-presenting capacity (44). Moreover, γδ T cells are usually potent cytotoxic effector cells which kill various tumor target cells independent of HLA restriction. Human Vγ9Vδ2 T cells recognize pyrophosphates accumulating in tumor cells exhibiting a dysregulated mevalonate metabolic pathway in a butyrophilin 3A-dependent manner (45). However, most γδ T cells also express the activating NKG2D receptor, which endows them with a TCR-independent second activation pathway *via* recognition of NKG2D ligands (e.g., MICA/B) on tumor cells. Based on their HLA-independent mode of target cell recognition, γδ T cells have recently attracted substantial interest as potential effector cells in cell-based cancer immunotherapy (46). This includes the perspective of using allogeneic γδ T cells from healthy donors since γδ T cells from the blood of tumor patients are sometimes difficult to expand *in vitro*. The experience of one of us (Zhinan Yin) with over 140 adoptive γδ T-cell transfers in more than 45 patients with different malignancies indicates that such γδ T-cell transfers are safe and are well tolerated.

In the murine system, genome-wide histone (H3) acetylation and methylation profiling have identified distinct molecular programs in interferon-γ versus IL-17 producing γδ T cells (47). It is also well established that epigenetic mechanisms regulate the chromatin accessibility of the TCR γ locus during intrathymic T cell development (48, 49). Currently, however, there is only limited information available as to how epigenetics contributes to the multifunctionality of human γδ T cells. We have performed a comprehensive analysis of peripheral blood αβ T cell subsets (CD4^+^, Treg, CD8^+^) and γδ T cells. In this ongoing work, we expect to obtain information on how γδ T cells differ from (subsets of) αβ T cells at the transcriptome and epigenetic level (Bhat et al., unpublished). Moreover, we have investigated the effects of the HDACi VPA on the Vγ9Vδ2 subset of human γδ T cells upon *in vitro* culture. VPA differentially modulated the expression of certain surface markers (notably CD86, CD54, and NKG2D) on γδ T cells compared with αβ T cells (50). For instance, NKG2D receptors on γδ T cells and their respective ligands on tumor cells were even more affected after VPA treatment (Bhat et al., under revision). We also observed that VPA induced the expression of a non-secreted isoform of IL-4 (IL-4δ13) which is known to have regulatory properties (51). Ongoing studies in our laboratories analyze the effects of VC on the *in vitro* differentiation of human γδ T cells. VC increases and stabilizes the expression of FoxP3 in transforming growth factor-β (TGF-β)-treated Vγ9Vδ2 T cells and augments the proliferative capacity of Vγ9Vδ2 T cells upon pyrophosphate-induced growth arrest (Kouakanou et al., to be published). RNA-seq and reduced representation bisulfite sequencing analyses of VC-treated human γδ T cells will provide insights how VC globally affects human γδ T-cell plasticity at the transcriptional and DNA methylation level. Though our study has been focused on the Vγ9Vδ2 subset, the effect of epigenetic drugs needs to be addressed in the context of distinct subsets of γδ T cells. Hence, the implication of epigenetic modulation needs to be investigated using different settings. Interestingly, we also found that TGF-β, usually considered as an immunosuppressive cytokine (52), can actually increase the cytotoxic activity of purified γδ T cells activated by pyrophosphate antigens in the presence of TGF-β (Peters et al., submitted). Thus, a variety of strategies are available to modulate the plasticity of human γδ T cells.

## How to Modulate the Anti-Tumor Potential of Multifunctional γδ T Cells?

Based on the outlined principles, we can envisage a multitude of approaches to enhance the cytotoxic anti-tumor activity of human γδ T cells, or to modulate their subset phenotype (Bhat et al., under revision), or to revert their detrimental activity (e.g., regulatory activity and/or high-PD-L1 expression of tumor-infiltrating γδ T cells) (53). DNMT inhibitors and HDACi already in clinical use modulate antigens relevant for γδ T-cell activation including NKG2D receptor and ligands (22, 26) and thus may increase the efficacy of adoptive γδ T-cell immunotherapy. Of special interest, however, are established and emerging new BET inhibitors. It will be important to find out whether BET inhibitors like JQ-1 can increase the functionality of *in vitro* expanded γδ T cells and eventually their persistence and anti-tumor activity similar to what has been described for tumor-reactive CD8^+^ T cells (36). BET inhibitors might also augment γδ T-cell immunotherapy *via* increasing the expression of NKG2D ligands on tumor cells (35). Furthermore, the recently reported BET inhibitor-mediated inhibition of PD-L1 expression on tumor cells [associated with improved anti-tumor immunity; Ref. (33)] might also extend to the inhibition of PD-L1 expression on tumor-infiltrating γδ T cells, which has been shown to restrain effective αβ T-cell responses in pancreatic oncogenesis (54). Last but not least, novel inhibitors have been developed selectively inhibiting the Brd interaction of CBP/EP300, which plays a crucial role in Treg biology (55). By dampening Treg activity, such small molecule inhibitors might also increase the efficacy of γδ T-cell immunotherapy in cancer patients. Overall, we have a plethora of strategies at hand to potentially increase the efficacy of γδ T-cell immunotherapy. The challenge is to design the best possible (pre-clinical and clinical) studies to identify efficacious synergistic strategies with acceptable adverse risk profile.

## Author Contributions

DK and JB wrote the manuscript. LK, CP, ZY, JB, and DK contributed to the discussion of the draft and made final corrections.

## Conflict of Interest Statement

DK is a member of the Scientific Advisory Board of Incysus, Ltd. The remaining co-authors declare that the research was conducted in the absence of any commercial or financial relationships that could be construed as a potential conflict of interest.
